# Disease-associated *DNA2* nuclease–helicase protects cells from lethal chromosome under-replication

**DOI:** 10.1093/nar/gkaa524

**Published:** 2020-06-16

**Authors:** Benoît Falquet, Gizem Ölmezer, Franz Enkner, Dominique Klein, Kiran Challa, Rowin Appanah, Susan M Gasser, Ulrich Rass

**Affiliations:** Friedrich Miescher Institute for Biomedical Research, CH-4058 Basel, Switzerland; Faculty of Natural Sciences, University of Basel, CH-4056 Basel, Switzerland; Friedrich Miescher Institute for Biomedical Research, CH-4058 Basel, Switzerland; Faculty of Natural Sciences, University of Basel, CH-4056 Basel, Switzerland; Friedrich Miescher Institute for Biomedical Research, CH-4058 Basel, Switzerland; Friedrich Miescher Institute for Biomedical Research, CH-4058 Basel, Switzerland; Friedrich Miescher Institute for Biomedical Research, CH-4058 Basel, Switzerland; Genome Damage and Stability Centre, School of Life Sciences, University of Sussex, Falmer, Brighton BN1 9RQ, UK; Friedrich Miescher Institute for Biomedical Research, CH-4058 Basel, Switzerland; Faculty of Natural Sciences, University of Basel, CH-4056 Basel, Switzerland; Friedrich Miescher Institute for Biomedical Research, CH-4058 Basel, Switzerland; Genome Damage and Stability Centre, School of Life Sciences, University of Sussex, Falmer, Brighton BN1 9RQ, UK

## Abstract

*DNA2* is an essential nuclease–helicase implicated in DNA repair, lagging-strand DNA synthesis, and the recovery of stalled DNA replication forks (RFs). In *Saccharomyces cerevisiae*, *dna2*Δ inviability is reversed by deletion of the conserved helicase *PIF1* and/or DNA damage checkpoint-mediator *RAD9*. It has been suggested that Pif1 drives the formation of long 5′-flaps during Okazaki fragment maturation, and that the essential function of Dna2 is to remove these intermediates. In the absence of Dna2, 5′-flaps are thought to accumulate on the lagging strand, resulting in DNA damage-checkpoint arrest and cell death. In line with Dna2’s role in RF recovery, we find that the loss of Dna2 results in severe chromosome under-replication downstream of endogenous and exogenous RF-stalling. Importantly, unfaithful chromosome replication in Dna2-mutant cells is exacerbated by Pif1, which triggers the DNA damage checkpoint along a pathway involving Pif1’s ability to promote homologous recombination-coupled replication. We propose that Dna2 fulfils its essential function by promoting RF recovery, facilitating replication completion while suppressing excessive RF restart by recombination-dependent replication (RDR) and checkpoint activation. The critical nature of Dna2’s role in controlling the fate of stalled RFs provides a framework to rationalize the involvement of *DNA2* in Seckel syndrome and cancer.

## INTRODUCTION

Dna2 is a conserved enzyme of DNA metabolism that comprises a nuclease domain with single-stranded DNA (ssDNA)-specific endonuclease activity fused to a superfamily 1 helicase domain with 5′-to-3′ translocation polarity (1–3). *DNA2* is essential for cell proliferation and embryonic development across species (2,4–6). Mutations within *DNA2* are associated with sensitivity to DNA replication stress (RS), genome instability, mitochondrial myopathy, and the primordial dwarfism disorder Seckel syndrome (7–10). Overexpression of *DNA2* is frequent in cancer cells and has been linked to poor patient prognosis (11,12). Mechanistically, *DNA2* has been implicated in DNA double-strand break (DSB) repair, checkpoint activation, Okazaki fragment processing, telomere homeostasis, centromeric DNA replication, and the recovery of stalled replication forks (RFs) (13). Pinpointing which of its functions are critical for cell proliferation is an important step in rationalizing the molecular pathologies associated with *DNA2*.

In *Saccharomyces cerevisiae*, the lethality caused by disruption of Dna2 is suppressed by concomitant loss of either the DNA helicase Pif1 (14) or the DNA damage checkpoint-mediator Rad9 (15). This indicates that Pif1 mediates the formation of DNA structures that elicit a Rad9-dependent checkpoint response in Dna2-defective cells, which in turn triggers cell-cycle arrest and *dna2*Δ inviability. Pif1 is known to remove obstacles to RF progression, including G-quadruplex structures, RNA-DNA hybrids (such as transcription-associated R-loops), and DNA-binding proteins (16,17). Moreover, Pif1 promotes homologous recombination-dependent DNA damage bypass (18) and replication restart at arrested or broken RFs by recombination-dependent replication (RDR) and break-induced replication (BIR), respectively (19,20).

Pif1-mediated toxicity in Dna2-defective cells has been attributed to excessive strand-displacement DNA synthesis by lagging-strand DNA polymerase δ (Pol δ), a reaction that Pif1 stimulates *in vitro* (21). During lagging strand synthesis, Pol δ extends the 3′-end of each Okazaki fragment such that the 5′-end of preceding Okazaki fragments is displaced and the RNA/initiator-DNA primer can be nucleolytically removed before ligation. It is proposed that extensive strand displacement could be promoted by Pif1, leading to long flaps bound by ssDNA-binding protein RPA, thereby creating a potent checkpoint-activating intermediate (22). While the 5′-flap endonuclease Rad27 (FEN1 in human) is thought to deal with the majority of Okazaki fragments, its activity is inhibited by RPA. In contrast, Dna2 is able to cleave RPA-covered DNA flaps *in vitro* (23,24). Thus, Dna2 is thought to play an essential housekeeping role by policing Okazaki fragment maturation, preventing a build-up of RPA-ssDNA and checkpoint-mediated cell-cycle arrest (15).

This Okazaki fragment processing model is not without caveats. Reconstitution experiments and the analysis of Okazaki fragments synthesized in budding yeast *in vivo* have indicated that the role of Dna2 in Okazaki fragment maturation is likely to be very limited (25–27). First, the extent of strand-displacement DNA synthesis on the lagging strand is largely unaffected by loss of Dna2 or Pif1 (27,28). There is also no evidence that DNA2 contributes to Okazaki fragment processing in human cells (9). Biochemically, nascent DNA flaps inhibit nucleotide incorporation by Pol δ, which favors instantaneous incision and flap removal by Rad27 once single-nucleotide or very short flaps are formed (29). Okazaki fragment maturation thus proceeds by nick translation without long flap intermediates. Polymerase idling, whereby Pol δ uses its 3′-to-5′ exonuclease activity to backtrack if flap removal by Rad27 is delayed, further limits the extensive growth of DNA flaps during Okazaki fragment processing (30). These observations collectively argue that DNA structures other than 5′-flaps at Okazaki fragments may be targeted by Dna2 to ensure cell survival.

From yeast to human, loss of Dna2 results in the accumulation of reversed RFs (6,31,32). These DNA four-way junctions arise under RS conditions at stalled and arrested RFs by the dissociation of the nascent leading and lagging strands from the parental template and their annealing with one another (33). Dna2 interacts dynamically with the DNA replication machinery at RFs via Ctf4 (And-1 in vertebrates) (9,34,35). In *Schizosaccharomyces pombe*, phosphorylation by the S-phase checkpoint kinase Cds1 ensures the presence of Dna2 at hydroxyurea (HU)-stalled RFs, where it is thought to counteract RF reversal by degrading the nascent DNA strands (6,36). Similarly, human DNA2 has been shown to resect stalled RFs in conjunction with Werner syndrome helicase WRN, resetting them to allow direct resumption of replication (31). In budding yeast, Dna2 accumulates in microscopically-visible nuclear foci in response to RS (37) and contributes to the processing of stalled replication intermediates (38), preventing the accumulation of post-replicative chromosome entanglements that impair sister chromatid separation unless they are cleaved by Holliday junction resolvase Yen1 in mitosis (39). These findings have established a physiologically-relevant contribution of the Dna2 nuclease–helicase to the completion of DNA replication that involves the processing of stalled RFs. Whether failed RF processing and recovery is sufficient to explain the inviability of cells upon loss of Dna2 is currently unclear. It is also unknown if and how Pif1 and Rad9-dependent checkpoint activation – the lethal event in *dna2*Δ cells – might arise from stalled RFs that have escaped the actions of Dna2.

Here, we show that RF-stalling results in incomplete chromosome replication when Dna2 is absent. Unresolved replication intermediates in Dna2-deficient cells are susceptible to Pif1, whose actions give rise to toxic DNA damage-checkpoint signaling in a manner dependent on its ability to promote homologous recombination-coupled DNA replication. These findings show that RF recovery is an indispensable Dna2 function and indicate the promiscuous use of homologous recombination-dependent replication restart as the mechanism of cell death in Dna2-deficient cells.

## MATERIALS AND METHODS

### Yeast strains and plasmids

*Saccharomyces cerevisiae* strains (Supplementary Table S1) were derived from BY4741 (40). If not stated otherwise, strains were cultured in YPAD medium at 30°C. The RS-sensitivity of the *dna2*Δ *pif1-m2* strain was complemented with plasmid-borne *DNA2* in vector pAG416GPD-ccbd (Addgene). The RS-sensitivity of *dna2*-*HD pif1-m2* cells was restored by expressing the sequence coding for the nuclear form of Pif1 (starting at amino acid residue M40) from a pYES-DEST52 vector (Invitrogen). Site-directed mutagenesis was used to generate *pif1*-*HD* (K264A). The *pif1-R3E* and *pif1-4a* alleles were introduced into the endogenous *PIF1* locus by Cas9-mediated mutagenesis (41). For microscopy, *YEN1* was cloned into pAG415GPD-ccbd-EGFP and NOP1-dsRED expressed from pWJ1321 (42).

### Cell viability, growth and drug-sensitivity assays

Doubling time, plating efficiency as a measure of strain viability, and cell growth in drop assays were determined as described (39). For liquid survival assays, overnight cultures were diluted to OD_600_ = 0.1–0.2, grown for 4 h in YPAD, synchronized in G1 using α-factor mating pheromone, washed, and then treated or not with 200 mM HU for 2 h in YPAD. Relevant dilutions were plated onto YPAD plates and colonies counted after 3–4 days. For colony size measurements, exponentially growing cells were plated on YPAD with or without HU. After 2 or 3 days, plates were imaged and analyzed using the Fiji image processing package with thresholding to exclude plate imperfections and fused colonies. Data were plotted using RStudio. Individual data points corresponding to single colonies are represented in a boxplot displaying the median value (black rectangle), the limits of the first and third quartile (lower and upper limits of the box), the most extreme data points that are less than 1.5× IQR from the limits of the first and third quartiles (whisker), and an approximation of the 95% confidence interval of the median (notches, median ± 1.57 × IQR/sqrt(*n*)). Mitotic time-course experiment, flow cytometry and analysis of Rad53-phosphorylation were carried out as described (39).

### Microscopy

For differential interference contrast images (DIC), yeast cells in exponential growth phase were harvested, fixed 5 min in 4% paraformaldehyde, washed 3 times in PBS (1 mM KH_2_PO_4_, 3 mM Na_2_HPO_4_, 150 mM NaCl), and attached to glass coverslips using concanavalin A (Sigma). Images were acquired with a Z1 Zeiss microscope, an AxioCam 506 mono camera, a Plan-APOCHROMAT 63×/1.4 DIC microscope oil objective, and Zen Blue 2012 software. For the observation of Yen1 foci, live cells were transferred to ibidi polymer-coated μ-Slides 4 Well imaging chambers (ibidi), and analyzed using an Olympus IX81 spinning disk confocal microscope equipped with an EM-CCD cascade II camera (Photometrics), a Yokogawa CSU-X1 scan head, and an ASI MS-2000 Z-piezo stage. The fluorophores were excited sequentially at 561 nm (dsRED) and 491 nm (EGFP) and the emitted light filtered with Semrock FF01-617/73-25 (dsRED) and Semrock FF01-525/40-25 (EGFP). Images were acquired using an oil-immersed PlanApo 100x/1.45 objective and Visiview software. For SIM experiments, cells were fixed in 4% paraformaldehyde, washed with PBS, and attached to a SIM-grade Zeiss 1.5 glass coverslip using concanavalin A. Image acquisitions were performed on a super resolution-SIM Elyra S.1 microscope (Zeiss) with a Plan-Apochromat 63×/1.4 NA objective lens, an EM-CCD camera iXon 885 (Andor Technology), and the ZEN software (Zeiss). Cells were fully sectioned into 60 slices at 0.1-nm intervals, with images taken at 60-ms exposures per slice with five rotations of the illumination grid. Image processing was performed with ZEN Black with the automatic settings and the ‘Raw scale’ option selected. For quantification, images were deconvolved using Huygens professional and the classic maximum-likelihood estimate algorithm with a signal-to-noise ratio of 5, automatic background estimation and 40 iterations. Thresholding and foci analysis were performed using the Fiji image processing package. Data were plotted using RStudio and the violin plot R package version 0.3.2 (https://github.com/TomKellyGenetics/vioplot). On violin plots, the black dot and the error bars represent the mean and the standard deviation of three independent biological replicates, respectively.

### Pulsed field gel electrophoresis (PFGE) and Southern blotting

Chromosomal DNA was analysed by PFGE (43). Yeast cells were harvested by centrifugation and washed in ice-cold 0.5 M EDTA. Cell pellets were suspended in Zymolyase buffer (50 mM NaH_2_PO_4_, 50 mM EDTA, 1 mM DTT) and embedded in 1% pulsed field-certified agarose (Bio-Rad) to form a plug. Plugs were treated 1 h at 37°C with 0.4 mg ml^−1^ Zymolyase (USBiological), overnight at 50°C with proteinase K (Eurobio) in 10 mM Tris–HCl, 50 mM EDTA, 1% *N*-lauroylsarcosinate (Sigma), and then washed with ddH_2_O. Genomic DNA was migrated through 1% agarose in 0.5× TBE (89 mM Tris–HCl, 89 mM boric acid, 2 mM EDTA) in a CHEF-DR II PFGE system (BioRad) maintained at 14°C, 6 V/cm, 60 s switch time for 15 h, followed by 90 s switch time for 9 h. Ethidium bromide-stained DNA was visualized using a GE Typhoon 9400 system. The DNA was then depurinated in 125 mM HCl solution, blotted onto Hybond-XL membrane (Amersham) by capillary transfer in NaOH, and fixed by baking at 80°C for 2 h. Membranes were blocked 1 h at 65°C with 20 ng ml^−1^ heat-denatured DNA (Sigma) in Church buffer (0.5 M sodium phosphate buffer, pH 7.5, 10 mM EDTA, 7% SDS) and probed overnight at 65°C for the chromosome of interest by Southern hybridization with sequence-specific, heat-denatured, radiolabeled DNA probes. After washing of the membranes, signals were recorded on phosphor-screens (Kodak) and visualized using a GE Typhoon 9400 system for quantification. Radiolabeled probes were generated by Klenow reaction: 200 ng of heat-denatured DNA template in labelling solution (50 mM Tris–HCl, 5 mM MgCl_2_, 0.2 M HEPES, pH 6.6, 150 μg ml^−1^ heat-denatured random hexadeoxyribonucleotides, 400 μg ml^−1^ bovine serum albumin, 25 μM GTP, 25 μM TTP, 0.3 μM [α-^32^P]ATP, 0.3 μM [α-^32^P]CTP) were incubated for 1 h at 37°C in presence of 5 U DNA Polymerase I Large Klenow Fragment (NEB). Radiolabeled DNA was purified on G-25 MicroSpin columns (GE Healthcare). Intensity calculations for Southern blots were performed using the Fiji image processing package. Signals for gel-resolved chromosomal DNA and DNA retained in the well were determined and background signal subtracted. The fraction of gel-resolved chromosomal DNA was calculated within each lane by dividing the signal for gel-resolved DNA by total DNA signal. For each experimental series, the migrating fraction of DNA following a single round of replication was expressed relative to the migrating fraction either in the respective G1 (maximal migration = 1) and HU samples (minimal migration = 0) or in the respective G1 (maximal migration = 1) and 40 min samples (minimal migration = 0) for the unperturbed S-phase progression.

## RESULTS

### DNA replication is not faithfully completed in unperturbed conditions when Dna2 is absent

**Figure 1.**
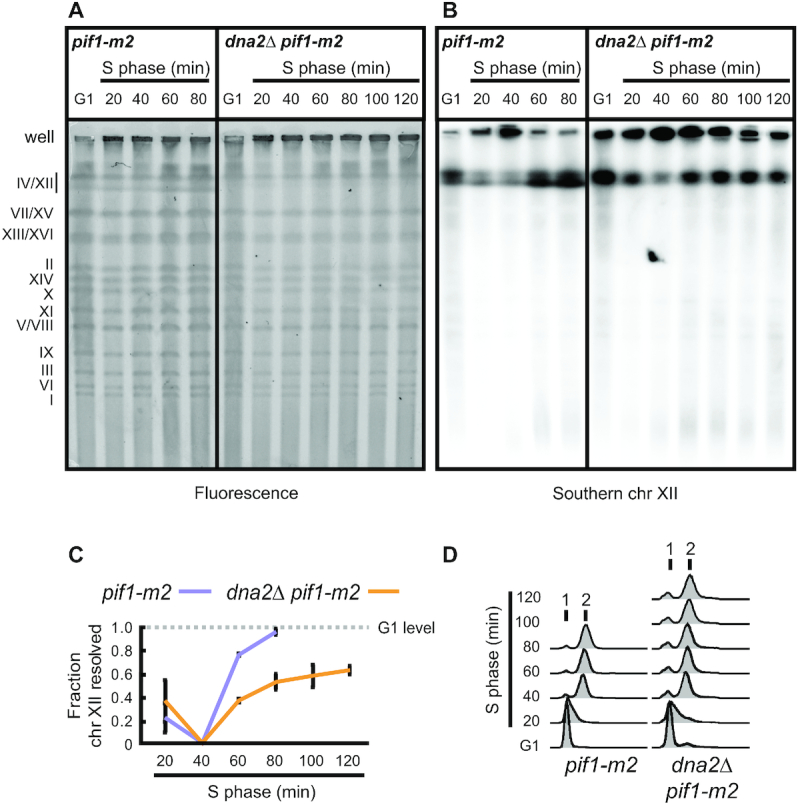
Dna2 is required for the completion of chromosome replication. (**A**) Representative PFGE experiments with the indicated strains, DNA stained by ethidium bromide. G1-synchronized cells were released into S phase in the presence of nocodazole to arrest cells prior to mitosis. At the indicated experimental stages, genomic DNA was analyzed for fully replicated (gel-resolved) chromosomes by PFGE. Gel-resolved DNA is labelled with chromosome numbers. (**B**) Southern blot analysis of the gel shown in panel A, probing for chromosome XII. (**C**) Quantification of Southern blots as shown in panel B to determine the fraction of gel-resolved chromosome XII. Data represent mean values ± SEM (*n* = 3 independent experiments). (**D**) Cell-cycle progression analysis by flow cytometry of cells collected as in panel A.

To address the impact of absent Dna2 on DNA replication, we characterized *dna2*Δ cells whose viability was restored by replacing *PIF1* with the *pif1-m2* allele (14). The *pif1-m2* allele results in the depletion of nuclear Pif1 while maintaining normal cell growth by expressing the mitochondrial form of Pif1 (44). Using pulsed-field gel electrophoresis (PFGE), we observed individual chromosomes before and after a single round of DNA replication. PFGE resolves fully replicated, linear chromosomes as discrete bands, while incompletely replicated chromosomes containing branched DNA intermediates remain in the wells of the gel. Cells were synchronized in G1 phase of the cell cycle using α-factor and released into S phase in medium containing nocodazole to prevent mitosis. Ethidium bromide staining after PFGE conspicuously showed a weaker increase of gel-resolved chromosomal DNA for *dna2Δ pif1-m2* cells passing through S phase compared to *pif1-m2* control cells in the region of chromosome XII (Figure 1A). Quantitative Southern blot analysis of chromosome XII from *pif1-m2* control cells showed full recovery of gel-resolved chromosomal DNA—indicative of complete replication—between 60 and 80 min after release into S phase. In contrast, genomic DNA of *dna2*Δ *pif1-m2* cells contained a much smaller fraction of gel-resolved material (approx. 50% reduction) and chromosome XII replication remained incomplete even 2 h after release into S phase (Figure 1B, C). Nevertheless, *dna2Δ pif1-m2* cells reached a 2N DNA content ∼40 min after release into S phase, with similar kinetics to *pif1-m2* control cells (Figure 1D). These data indicate that while *dna2Δ pif1-m2* cells are proficient in bulk DNA synthesis, chromosome XII replication is not faithfully completed. Chromosome XII contains the rDNA array, which is highly transcribed and replicated in a unidirectional manner (45,46). This makes chromosome XII particularly challenging to replicate and stochastic RF arrest is less likely to be compensated by fork convergence compared to other regions of the genome. The overt underreplication of chromosome XII in unperturbed conditions is thus consistent with a reduced capacity of RF recovery in the absence of Dna2.

### Transient replication stress results in genome-wide under-replication in the absence of Dna2

**Figure 2.**
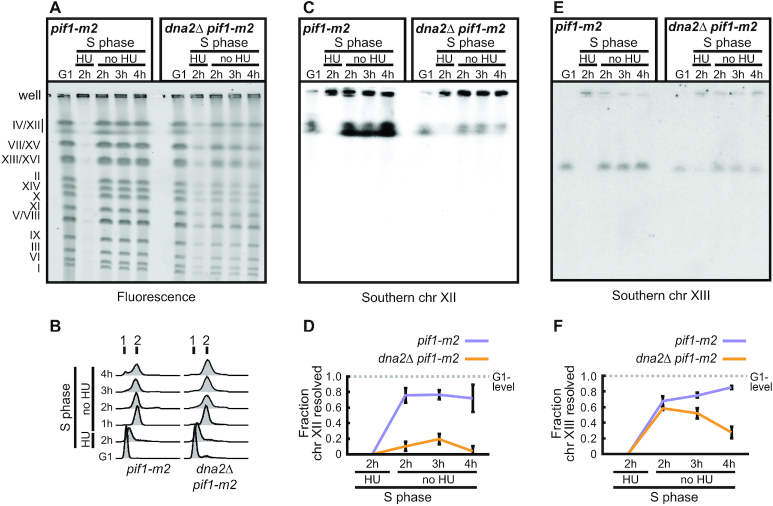
Transient replication arrest induces under-replication in the absence of Dna2. (**A**) Representative PFGE of the indicated strains treated with HU, DNA stained by ethidium bromide. G1-synchronized cells were released into medium containing 200 mM HU for 2 h, followed by drug wash-out and incubation in HU-free medium containing nocodazole to prevent mitosis. At the indicated stages, genomic DNA was analyzed for fully replicated (gel-resolved) chromosomes by PFGE. Gel-resolved DNA is labelled with chromosome numbers. (**B**) Cell-cycle progression analysis by flow cytometry of cells collected for the experiment shown in panel A. (**C**) Southern blot analysis of a gel obtained as in panel A, probing for chromosome XII. (**D**) Quantification of Southern blots as shown in panel C to determine the fraction of gel-resolved chromosome XII. Data represent mean values ± SEM (*n* = 3 independent experiments). (**E**) Southern blot analysis of a gel obtained as in panel A, probing for chromosome XIII. (**F**) Quantification of Southern blots as shown in panel E to determine the fraction of gel-resolved chromosome XIII. Data represent mean values ± SEM (*n* = 3 independent experiments).

To further address whether unfaithful chromosome replication in *dna2Δ pif1-m2* cells is linked to an inappropriate response to stalled RFs when Dna2 is absent, we transiently exposed *dna2Δ pif1-m2* cells to exogenous RS. Cells were synchronized in G1 using α-factor and released into S phase in the presence of 200 mM HU to arrest RF progression. After 2 h, HU was removed to allow cells to continue replication while nocodazole was added to the medium to prevent mitosis. Genomic DNA of cells arrested in G1 phase and released into S phase for increasing amounts of time was analyzed by PFGE. Compared to non-RS conditions, replication completion in the absence of Dna2 appeared more dramatically impaired across chromosomes after ethidium bromide staining (Figure 2A), despite *dna2*Δ *pif1-m2* cells resuming DNA synthesis and progressing through bulk DNA synthesis with kinetics similar to *pif1-m2* control cells after HU-removal (Figure 2B). Quantitative Southern blot analysis revealed a severe defect in completing chromosome XII replication after acute RS-exposure for *dna2*Δ *pif1-m2* cells compared to *pif1-m2* control cells (Figure 2C, D). To address whether this chromosome under-replication phenotype may be mediated by residual nuclear Pif1 in *dna2*Δ *pif1-m2* cells, we performed an analysis of genomic DNA from *dna2*Δ *pif1*Δ cells using PFGE. Again, we observed significant chromosome XII under-replication (Supplementary Figure S1A, B). Similar results were obtained for *dna2*Δ *rad9*Δ cells (Supplementary Figure S1C, D). These findings confirm that Dna2 is needed to promote replication completion following transient RF arrest regardless of the presence or complete absence of Pif1, and that this requirement for Dna2 is fully independent of any actions of Pif1, including excessive strand displacement on the lagging strand.

We next analyzed chromosome XIII, which lacks rDNA repeats, by quantitative Southern blotting. Compared to chromosome XII, we detected a less dramatic, yet marked shortfall in gel-resolved material for *dna2*Δ *pif1-m2* cells after passage through S phase (Figure 2E, F). These findings indicate that, in response to transient RF arrest, Dna2 is required genome-wide to ensure chromosome replication is faithfully completed.

### Transient replication arrest is cell-lethal in the absence of Dna2

**Figure 3.**
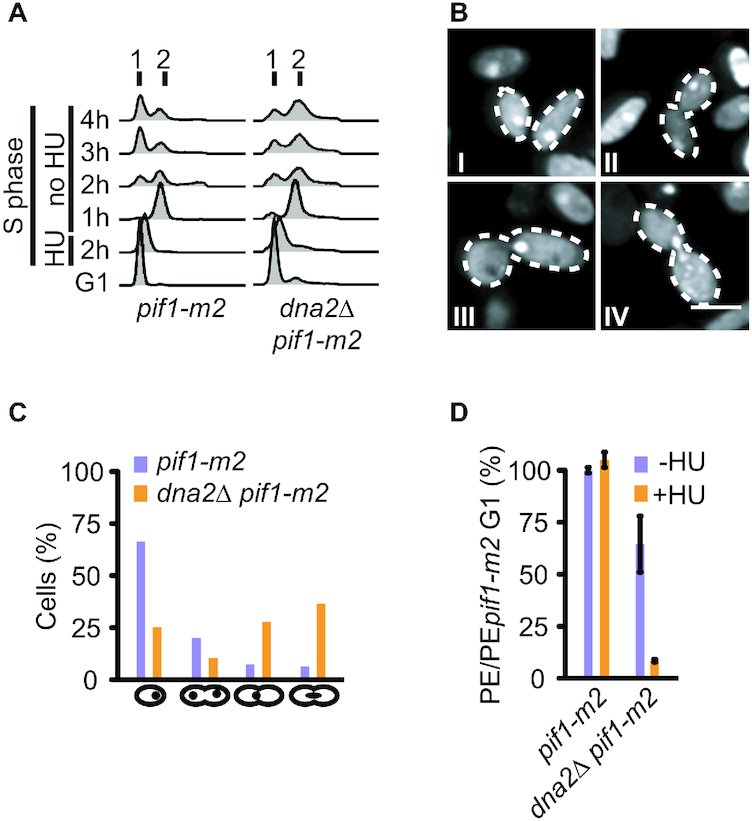
Transient RF arrest in the absence of Dna2 is cell-lethal. (**A**) Cell**-**cycle progression analysis by flow cytometry of the indicated strains synchronized in G1, treated with 200 mM HU for 2 h and released for 4 h into a drug-free medium. (**B**) Representative images of *dna2*Δ *pif1-m2* cells treated as in panel A showing single-nucleated cells (I), a double-nucleated cell in G2 (II), a G2 cell with a nucleus at the bud neck (III), and an anaphase cell with an elongated nucleus spanning the bud neck (IV). Scale bar, 5 μm. (**C**) Quantification of cells (n ≥ 110 cells per strain) observed as in panel B. (**D**) Cell viability of the indicated strains, assessed by plating efficiency (PE) after synchronization in G1, removal of α-factor, and treatment or not with 200 mM HU for 2 h. Data expressed relative to *pif1-m2* cells as mean values ± SD (*n* = 3 independent experiments).

While removal of Pif1 restores viability to *dna2*Δ cells, *dna2*Δ *pif1* double-mutant cells exhibit sensitivity to chronic RS (14) (Supplementary Figure S2A, B). Our observations of incomplete chromosome replication in *dna2*Δ *pif1* cells after transient RS-exposure further suggested that Dna2’s role in RF recovery is indispensable in the immediate response to acute RF arrest. Consistent with this notion, we found that the release of *dna2*Δ *pif1-m2* cells into S phase in the presence of 200 mM HU for 2 h and subsequent recovery in drug-free medium resulted in the accumulation of cells at the G2/M boundary with a 2N DNA content within one cell cycle (Figure 3A). Microscopic inspection showed that *dna2Δ pif1-m2* cells accumulated as large-budded G2/M cells with either a single nucleus or an elongated nucleus spanning the bud neck, indicative of physical impediments to segregation in anaphase cells at sites of unfinished chromosome replication (Figure 3B, C). Compared to *pif1-m2* control cells, the viability of *dna2Δ pif1-m2* cells dropped dramatically as a consequence of the transient RF arrest (Figure 3D). These results indicate that Dna2-dependent RF recovery is essential to ensure complete chromosome replication, faithful chromosome segregation, and cell viability after a transient replication block.

### Resolution of under-replicated DNA by Yen1 is indispensable for cell viability when Dna2 is absent

**Figure 4.**
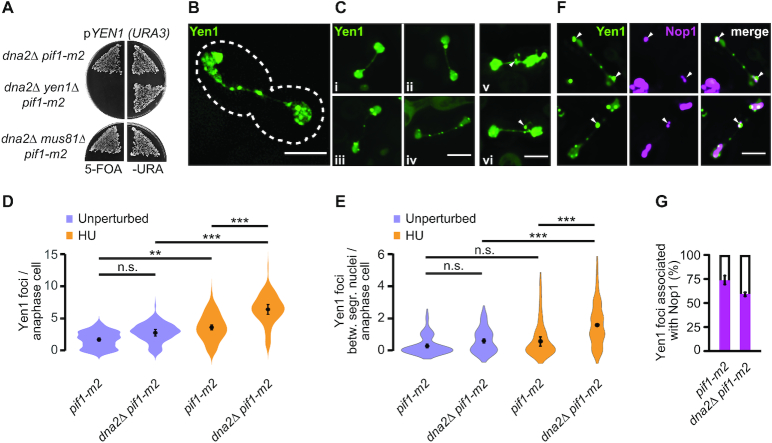
The resolution of under-replicated chromosomes by Yen1 is indispensable in the absence of Dna2. (**A**) Requirement for *YEN1* and *MUS81* in *dna2*Δ *pif1-m2* cells. Cells contained a Yen1-expressing plasmid (*pYEN1*). Lack of growth on 5-FOA indicates an inviable genotype. (**B**) SIM image of an anaphasic *pif1-m2* control cell expressing Yen1-EGFP (green). Scale bar, 5μm. (**C**) Representative images of anaphasic *pif1-m2* (i-ii) and *dna2*Δ *pif1-m2* (iii-vi) cells expressing Yen1-EGFP. Images v and vi show Yen1 foci located on a loop-like structure indicative of rDNA (arrowheads). Scale bar, 5 μm. (**D**) Quantification of Yen1-EGFP foci per anaphase cell in the indicated strains. Unperturbed samples are from exponentially growing cultures. For HU samples, cells were synchronized in G1, treated with 200 mM HU for 2 h and released into drug-free medium. All data represented as violin plots with the mean ± SD (*n* = 3 independent experiments, ≥ 99 cells observed per strain and condition). Statistical analysis by one-way analysis of variance (Anova) and post-hoc Tukey multiple comparison test (n.s., non-significant; **P< 0.01; ***P< 0.001). (**E**) Quantification as in panel D, focused on Yen1 foci between segregating masses of DNA. (**F**) Representative images of anaphasic *dna2*Δ *pif1-m2* cells with Yen1-EGFP (green) and Nop1-dsRED (magenta) foci on the separating DNA masses (top panel, arrowheads) and within the anaphase tube connecting them (bottom panel). Cells were synchronized in G1, treated with 200 mM HU for 2 h and released into drug-free medium. Scale bar, 5 μm. (**G**) Quantification of Yen1 and Nop1 co-localization, determined as in panel F. Data are represented as mean values ± SD (*n* = 3 independent experiments, ≥61 cells scored per strain and condition).

The Holliday junction resolvase Yen1 is a structure-specific nuclease best known for its role in the processing of branched homologous recombination intermediates (47,48). We have shown previously that Yen1 also resolves persistent replication intermediates in *dna2* hypomorphic cells exposed to RS, thereby facilitating chromosome segregation (39). While Yen1 exhibits functional redundancy with the structure-specific nuclease Mus81-Mms4 in the resolution of recombination intermediates, the role in removing post-replicative chromosomal DNA-links derived from stalled RFs in *dna2*-mutant cells is unique to Yen1. Given the overt under-replication that we detected in *dna2*Δ *pif1-m2* cells, we hypothesized that Yen1 may become essential in these cells, perhaps even in unperturbed conditions. This is indeed the case, as we found that deleting *YEN1* causes lethality in *dna2*Δ *pif1-m2* cells (Figure 4A). Similarly, loss of *YEN1* was not tolerated in *dna2*Δ *rad9*Δ cells (Supplementary Figure S3). In contrast, *dna2*Δ *pif1-m2* cells tolerated the deletion of *MUS81* (Figure 4A). We interpret these data to suggest equivalence between the response induced by exogenous RS in *dna2* hypomorphic cells (39) and the response to intrinsic replication perturbations in *dna2*Δ cells. In each case, dysfunctional RF recovery results in a significant burden of unresolved replication intermediates that require resolution by Yen1 for cell survival.

To analyze Yen1 dynamics, we used super-resolution structured illumination microscopy (SIM), monitoring a fully functional version of endogenous Yen1 (39,49) tagged with enhanced green-fluorescent protein (EGFP). Yen1 is activated at the onset of mitosis, when the protein accumulates in its nucleolytically-active form in the nucleus (49,50). We detected Yen1-EGFP in discrete foci and as a diffuse signal tracing the segregating nuclei and the tube-like structure that connects them in anaphase (Figure 4B, C). We observed that cells tend to form a greater number of Yen1 foci if Dna2 is absent (average number of Yen1-EGFP foci of 2.7 ± 0.5 for *dna2*Δ *pif1-m2* cells compared to 1.7 ± 0.3 for *pif1-m2* control cells), although this increase was not statistically significant in unperturbed conditions (Figure 4D). Treatment with 200 mM HU in the preceding S phase led to a marked increase of Yen1 foci in anaphase in both *pif1-m2* control and *dna2*Δ *pif1-m2* cells. This is consistent with a build-up of structures bound by Yen1 following RS. Importantly, under RS-conditions, the absence of Dna2 correlated with a significant increase in Yen1-EGFP foci compared to control (average number of Yen1-EGFP foci of 6.4 ± 0.8 for *dna2*Δ *pif1-m2* cells compared to 3.6 ± 0.4 for *pif1-m2* control cells, *P* < 0.001). Moreover, while Yen1 foci mainly localized to the bulk of the separating masses of DNA in *pif1-m2* control cells, there was a marked increase of foci along the bridge connecting them in *dna2*Δ *pif1-m2* cells, in particular after HU-treatment (average number of Yen1-EGFP foci on bridges of 1.6 ± 0.1 for *dna2*Δ *pif1-m2* cells compared to 0.5 ± 0.3 for *pif1-m2* control cells, *P* < 0.001) (Figure 4E).

In some cases, Yen1-EGFP assembled into foci on circular structures that emanated from the bridge between the separating nuclei (Figure 4C). These structures were suggestive of rDNA-loops (51,52), consistent with previous findings in unperturbed conditions that Yen1 mainly accumulates within the rDNA (53). To address the co-localization of RS-induced Yen1 foci with the rDNA, we expressed nucleolar protein Nop1 (54) tagged with RFP in *pif1-m2* cells and *dna2*Δ *pif1-m2* cells. The majority of Yen1 foci (73 ± 4%) colocalized with, or bordered, Nop1-marked rDNA in *pif1-m2* control cells. While *dna2*Δ *pif1-m2* cells contained a similar number of Yen1-EGFP foci associated with Nop1-RFP, co-localization decreased to 58 ± 2% due to an increase in Yen1 foci not marked by Nop1 (Figure 4F, G). These finding are consistent with the notion that Yen1 target structures are predominantly associated with the rDNA (53) and elevated elsewhere in the genome in *dna2*Δ *pif1-m2* cells after RS, in line with our PFGE analyses showing a genome-wide replication defect in the absence of Dna2.

Taken together, these findings provide further evidence for a key role of Dna2 in the recovery of stalled RFs. In *dna2*Δ *pif1-m2* cells, Yen1 fulfils an essential function by resolving persistent replication intermediates that arise at stalled RFs not properly processed by Dna2.

### Unresolved replication intermediates in *dna2* helicase-defective cells expose to Pif1-mediated DNA damage checkpoint activation

Although chromosome under-replication in *dna2*Δ *pif1* double-mutant cells turned out to be significant, cell viability is maintained by Yen1 unless exogenous RS is applied. This is perhaps not surprising, given that lethality in *dna2*Δ cells in unperturbed conditions requires the presence of Pif1 (14), whose actions lead to Rad9-dependent checkpoint activation and terminal cell-cycle arrest (15). This raised the question whether Pif1/Rad9-dependent toxicity in Dna2-deficient cells arises as a consequence of an unfaithful response to RF-stalling and chromosome under-replication or, as previously proposed, through a defect in Okazaki fragment processing (15). To address this, we turned to *dna2* hypomorphic cells harboring the *dna2-2* allele (55). The *dna2-2* allele encodes Dna2 R1253Q, which is a nuclease-proficient and helicase-deficient Dna2 (39), and will henceforth be referred to as *dna2-HD*, for helicase-dead. Like *dna2*Δ *pif1* cells, *dna2*-*HD* cells are RS-sensitive and accumulate unresolved replication intermediates that require processing by Yen1 (39), but unlike *dna2*Δ cells, they tolerate the presence of nuclear Pif1. This is likely due to the intact nuclease activity of this helicase-dead Dna2, which provides partial protection from RS in the absence of helicase activity, indicating that Dna2-dependent RF recovery is compromised but not abolished (Supplementary Figure S4A). Thus, *dna2*-*HD* cells allow us to compare the response to RS in a Dna2-defective setting in the presence and absence of Pif1.

**Figure 5.**
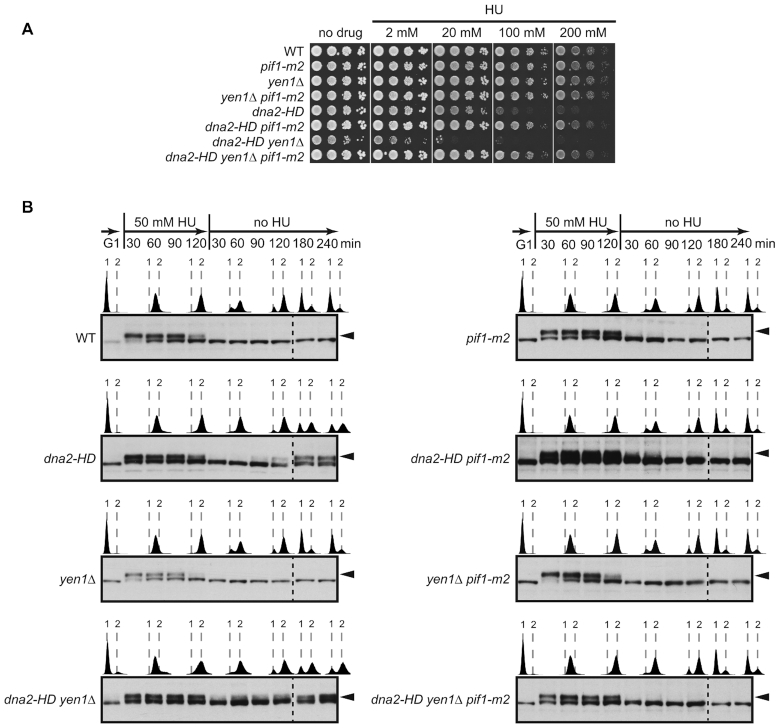
Incomplete replication upon Dna2 dysfunction gives rise to Pif1-mediated DNA damage checkpoint activation. (**A**) Drop assays with the indicated strains on drug-free and HU-containing plates to assess growth and RS-sensitivity. (**B**) Mitotic time-course experiments with transient RS-treatment of the indicated strains. Cells, synchronized in G1, were released into medium containing 50 mM HU for 2 h, followed by drug wash-out and incubation in drug-free medium with α-factor to prevent entry into a second S phase. Checkpoint activation was monitored by Western blot analysis of Rad53 hyperphosphorylation (black arrowheads). The progression of DNA replication was monitored by flow cytometry (1 and 2 N DNA content are indicated).

Dna2 helicase-defective cells exhibit a number of growth defects and fail to progress properly through the cell cycle, even in unperturbed conditions (39). These phenotypes are exacerbated by the deletion of *YEN1*, but were strongly suppressed upon introduction of the *pif1-m2* allele. Thus, *dna2-HD pif1-m2* and *dna2*-*HD yen1*Δ *pif1-m2* cells exhibited near wild-type doubling times, viability, and cell-cycle progression at the G2/M boundary (Supplementary Figure S4B-D). The *pif1-m2* allele also suppressed background DNA damage-checkpoint activation and the accumulation of G2/M-arrested cells with a large-budded morphology that characterizes *dna2-HD* and *dna2*-*HD yen1*Δ cultures (Supplementary Figure S4E, F). Under RS conditions, we observed that the *pif1-m2* allele conferred increased RS-resistance to *dna2-HD* and *dna2*-*HD yen1*Δ cells (Figure 5A), which is in line with previous findings (14). However, *dna2-HD pif1-m2* and, to a greater extent, *dna2*-*HD yen1*Δ *pif1-m2* cells remain RS-sensitive compared to *pif1-m2* and *yen1*Δ *pif1-m2* control cells, which is revealed by colony size measurements (Supplementary Figure S4G, H). Next, we sought to clarify whether the Pif1-independent defect in RF recovery and faithful chromosome replication in Dna2-defective cells and the strong Pif1-dependent toxicity arising in these cells are connected.

We have shown previously that the accumulation of unresolved replication intermediates when *dna2-HD* cells are transiently exposed to RS is accompanied by an unusual, post-replicative DNA damage-checkpoint response. This checkpoint response is Rad9-dependent and occurs at the G2/M boundary independently of cytokinesis (39). We asked the question whether Pif1 is involved in this unscheduled checkpoint activation. G1-synchronized cells were released into S phase in the presence of 50 mM HU for 2 h, before removing HU from the medium and incubating the cells for a further 4 h. Under these conditions, cells can complete one cell cycle and are held in the next G1 phase by addition of α-factor to the medium. As expected (39), *dna2-HD* and *dna2-HD yen1*Δ cultures showed a normal response to HU by activating the S-phase checkpoint, which was silenced upon removal of HU (Figure 5B). Then, as cultures reached the end of bulk DNA synthesis, indicated by a 2N cellular DNA content, wild-type cells started to divide ∼2 h after HU-removal. In contrast, *dna2-HD* and *dna2-HD yen1*Δ cells arrested at the G2/M boundary and exhibited renewed Rad53 phosphorylation (Figure 5B). Strikingly, depletion of nuclear Pif1 abolished this unscheduled, HU-induced G2/M checkpoint signaling in *dna2-HD pif1-m2* and *dna2*-*HD yen1*Δ *pif1-m2* cells and progression into mitosis was no longer delayed (Figure 5B). Pif1 therefore drives G2/M DNA damage-checkpoint activation provoked by transient RS in Dna2 helicase-defective cells, linking Pif1/Rad9-mediated toxicity to unresolved replication intermediates.

Collectively, these results strongly suggest Pif1 acts on stalled RFs that are not properly resolved by Dna2. This gives rise to DNA damage checkpoint-activating DNA structures, cell-cycle arrest, and cytotoxicity in Dna2 helicase-deficient cells.

### Pif1/Rad9-mediated toxicity in Dna2 helicase-defective cells results from inappropriate recombination-dependent replication

**Figure 6.**
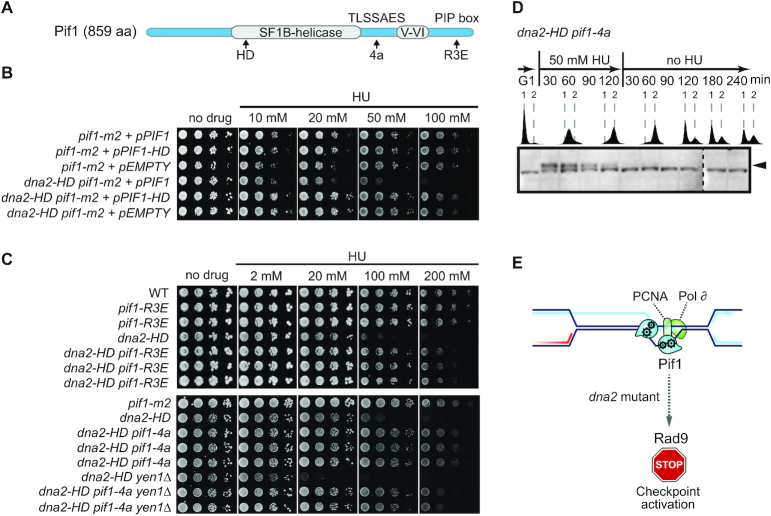
Pif1-toxicity in Dna2-defective cells maps to homologous recombination-coupled DNA replication. (**A**) Schematic representation of Pif1 showing the bi-partite helicase domain (grey), and a TLSSAES phosphorylation motif and PCNA-interacting peptide (PIP box) required for homologous recombination-coupled DNA replication. Mutations introduced into these domains are denoted below, see text for details. HD, helicase-dead. (**B**) Drop assays of serial dilutions on drug-free and HU-containing medium of the indicated strains expressing wild-type Pif1 (+*pPIF1*), a helicase-dead version of Pif1 (+*pPIF1-HD*), or containing the empty vector. (**C**) Drop assays with the indicated strains on drug-free and HU-containing medium assessing growth and sensitivity to RS in the presence or absence of the *pif1-R3E* and *pif1-4a* alleles at the endogenous *PIF1* locus in multiple independently created strains. (**D**) *dna2-HD pif1-4a* cells were assessed for unscheduled G2/M DNA damage-checkpoint activation following transient exposure to RS as in Figure 5, panel B. (**E**) The mutational analysis of Pif1 strongly implicates homologous recombination-coupled DNA replication at stalled RFs as the pathway generating toxicity by unscheduled DNA damage-checkpoint activation in Dna2-mutant cells.

To gain insight into how Pif1 mediates the generation of structures that elicit a Rad9-dependent checkpoint response in the presence of unresolved replication intermediates in Dna2 helicase-deficient cells, we analyzed the effect of well-characterized Pif1 mutants (Figure 6A). First, we disrupted the catalytic activity of Pif1, comparing *dna2-HD pif1-m2* cells expressing wild-type Pif1 or helicase-dead variant Pif1 K264A (*pif1-HD*) (56). Only the expression of catalytically active Pif1 sensitized the cells to RS (Figure 6B), showing that Pif1’s helicase activity is required for the toxicity induced upon RF-stalling in cells with compromised Dna2 activity.

Next, we targeted an interaction between Pif1 and the replicative sliding clamp PCNA (20). Structure-function analyses have shown that a Pif1-R3E mutant (with point mutations I817R, M820R, L821R and R823E) loses the ability to bind PCNA, to co-localize with homologous recombination-mediator Rad52 following RS (18), and to mediate homologous recombination-coupled DNA synthesis via BIR (57). Strikingly, introduction of the *pif1-R3E* allele into the endogenous *PIF1* locus rendered *dna2-HD* cells markedly more resistant to RS (Figure 6C). The phenotypic suppression was similar to that achieved by depletion of nuclear Pif1 (see Figure 5A).

Homologous recombination-coupled replication also depends on a TLSSAES phosphorylation motif within the C-terminal domain of Pif1 (58). Disruption of TLSSAES by exchanging all phosphorylatable threonine and serine residues for alanine (Pif1-4A mutant with T763A, S765A, S766A and S769A) renders Pif1 defective for homologous recombination-coupled replication, while other cellular functions of Pif1 are preserved (58,59). Introduction of the *pif1-4A* mutation into the endogenous *PIF1* locus resulted in strong suppression of the RS-sensitivity observed for *dna2-HD* and *dna2-HD yen1*Δ cells (Figure 6C). Importantly, disruption of the TLSSAES motif also suppressed the unscheduled DNA damage-checkpoint response and G2/M arrest exhibited by *dna2-HD* cells in response to RF-stalling (Figure 6D, see also Figure 5B) and restored viability to *dna2*Δ cells (Supplementary Figure S5).

Together, these results indicate that Dna2 is critically required to control the fate of stalled RFs. Blocked RFs that haven’t been properly processed by Dna2 are subject to Pif1 activity, ultimately leading to DNA damage-checkpoint activation in *dna2*-mutant cells and cell death. The suppression of these phenotypes by Pif1 mutations that converge on blocking homologous recombination-coupled DNA synthesis imply that the toxicity caused by Pif1/Rad9 when Dna2 function is compromised is a direct consequence of unfaithful RF recovery and inappropriate replication restart by RDR (Figure 6E).

## DISCUSSION

Precisely why the multifunctional genome caretaker *DNA2* is essential for viability has remained unknown. Here, we identify Dna2’s role in RF recovery as essential for cell survival. We propose that upon Dna2 dysfunction, stalled RFs engage in excessive homologous recombination-coupled restart reactions, resulting in the accumulation of DNA checkpoint-activating intermediates, terminal cell-cycle arrest, and loss of cell viability. This provides a conceptual alternative to impaired Okazaki fragment processing as the cause of inviability of *dna2-*null cells.

Loss of Dna2 resulted in unfaithful chromosome replication and a significant burden of unresolved replication intermediates, in particular within difficult-to-replicate regions of the genome (Figure 1). Transient exposure to RS exacerbated this phenotype genome-wide and within one cell cycle, indicating that a dysfunctional response to stalled RFs underpins the chromosomal under-replication phenotype of *dna2*-null cells (Figure 2). Unresolved replication intermediates subsequently jeopardize mitotic progression (Figure 3), but, if not excessive, can be resolved by the structure-specific nuclease Yen1 to allow viable mitotic exit. Consequently, Yen1 becomes essential for cell survival in the absence of Dna2 (Figure 4 and Supplementary Figure S3). Yen1 localized to DNA structures arising from RS, and, in absence of Dna2, preferentially bound chromatin bridges connecting the DNA masses of segregating anaphase nuclei (Figure 4). We interpret this as a manifestation of Yen1’s engagement with chromosome entanglements that result from dysfunctional RF recovery and under-replication, subsequently impairing chromosome segregation.

These observations underscore the physiological importance of the RF-recovery role of Dna2 (6,31,36,39,60) and raised the question whether the lethality that Pif1 and Rad9 induce in *dna2*-null cells (14,15) might be linked to the accumulation of unresolved replication intermediates. We determined that, like Pif1/Rad9-mediated lethality in *dna2*Δ cells, RS-induced toxicity in hypomorphic *dna2-HD* cells is largely mediated by Pif1/Rad9. In these cells, Pif1 drives unscheduled DNA damage-checkpoint signaling following transient exposure to RS (Figure 5). These findings place Pif1/Rad9-dependent toxicity downstream of RF-stalling. Notwithstanding the possibility of an involvement of multiple Pif1 functions, our mutational analysis of Pif1 strongly implicates the resumption of DNA synthesis by homologous recombination-dependent pathways such as BIR and RDR (61) in Pif1-mediated toxicity (Figure 6). At broken RFs, where there is a single-ended DSB, homologous recombination proteins assemble at the DSB and catalyze strand-invasion of an intact chromosome to prime DNA synthesis within a displacement-loop (D-loop). BIR then proceeds by bubble-migration with uncoupled leading and lagging-strand DNA synthesis. A similar process, RDR, occurs at arrested but unbroken RFs (62–64). In this case, it is likely that the regressed arm of reversed RFs provides the recombination substrate, which is used for invasion of the parental duplex ahead of the site of fork reversal with subsequent D-loop DNA synthesis. Pif1 is essential to drive DNA synthesis in the context of a D-loop, which often covers many thousands of nucleotides (19,20,65). At D-loops, Pif1 binds PCNA, and loss of PCNA interactions renders Pif1 BIR-defective (57). It has also been shown that the ability of Pif1 to promote BIR depends on a DNA damage-responsive TLSSAES phosphorylation site (58,59). Here we find that, like BIR/RDR, RS-sensitivity and RS-induced G2/M-checkpoint activation in Dna2 helicase-defective cells requires the catalytic activity of Pif1, its interaction with PCNA, and the TLSSAES phosphorylation motif (Figure 6).

Potential substrates for RDR, reversed RFs, have been shown to accumulate in Dna2-depleted cells (6,31,32). This may shift the balance of RF recovery from direct replication resumption mediated by Dna2-dependent RF processing when the replication impediment is resolved or removed to Pif1-mediated RDR. RDR *per se* is not expected to be toxic. Indeed, RDR could mitigate under-replication problems caused by the absence of Dna2. However, D-loop progression during BIR and RDR has been shown to be unstable and the nascent DNA strand undergoes frequent cycles of dissociation and re-annealing with the template (66–69). If used promiscuously, RDR may thus become a significant source of checkpoint-activating intermediates by way of D-loop collapse, potentially leaving nascent strands of extended length permanently exposed as ssDNA to cause cell-cycle arrest and lethality in Dna2-deficient cells.

**Figure 7.**
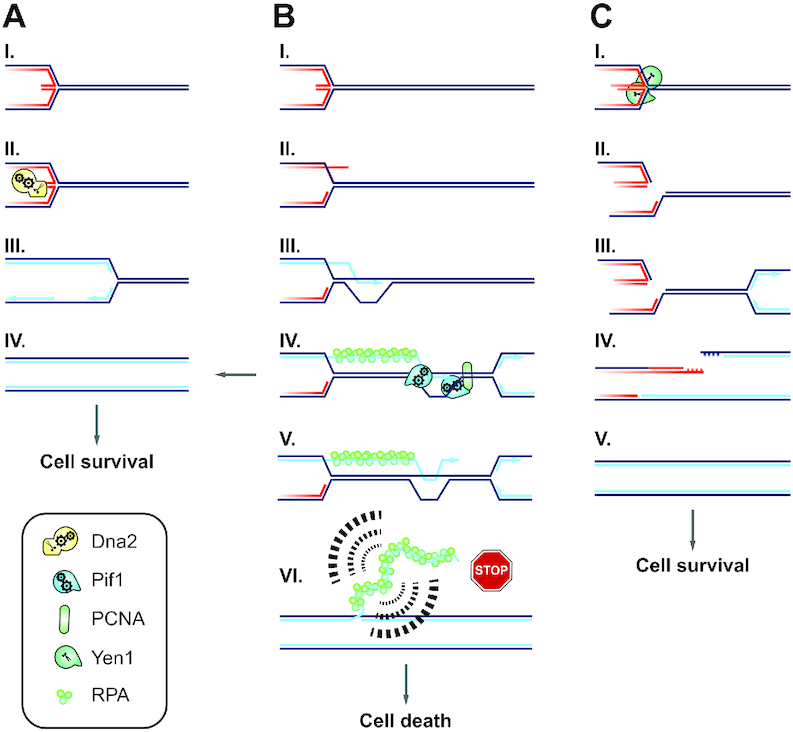
Model of Dna2 fulfilling an essential role at stalled RFs by promoting RF recovery and suppressing inappropriate restart by RDR. (**A**) Dna2 counteracts RF reversal at stalled RFs (I) by degrading nascent ssDNA or unwinding and degrading the regressed dsDNA arm (II) (6,31). This enables direct resumption of RF progression (III) and promotes the completion of chromosome duplication (IV). (**B**) Stalled RFs left unprocessed by Dna2 (I) are subject to alternative restart by RDR (II), leading to the formation of a D-loop (III). Pif1 promotes homologous recombination-coupled DNA replication (19,20) by binding PCNA and stimulating DNA synthesis in the context of a D-loop (57) (IV). RDR can facilitate replication completion and cell survival (arrow pointing left). However, migrating D-loops are characterized by frequent nascent strand dissociation (V) (66–69). D-loop collapse and passage of a conventional RF may cause nascent RDR strands to become permanently exposed as ssDNA, resulting in checkpoint-activating structures with long ssDNA branches that conform to Pif1-dependent intermediates recently observed in Dna2-depleted cells (VI) (32). This explains why Dna2 is essential: In the absence of Dna2, inappropriate RDR results in toxic levels of RPA-covered ssDNA, causing DNA damage-checkpoint activation and terminal cell-cycle arrest. (**C**) In the absence of Dna2 and Pif1, replication remains incomplete, but because stalled RFs are unable to undergo excessive RDR, the DNA damage checkpoint remains silent and cells enter mitosis. This activates Yen1 (I), enabling the resolution of chromosome entanglements at persistent replication intermediates (II). The DNA repair steps downstream of Yen1-cleavage remain to be determined. It is conceivable that arrival of a converging RF (III) facilitates replication of the unbroken sister chromatid and DSB repair on the sister chromatid cleaved by Yen1 (IV), thereby mediating replication completion (V).

Is there direct evidence for extended ssDNA in cells when Dna2 absent? The aberrant DNA intermediates that accumulate in cells after acute depletion of Dna2 have recently been evaluated by electron microscopy. The extent of their accumulation was strongly dependent on replication speed and nuclear Pif1 (32). The most prominent structures that accumulated in a Pif1-dependent manner consisted of linear double-stranded DNA (dsDNA) with a single ssDNA branch. One explanation for the occurrence of these dsDNA/ssDNA structures is that, in absence of Dna2, extensive DNA strand-displacement synthesis occurs on the lagging strand and remains unresolved due to improper flap-control during Okazaki fragment maturation (32). The vast majority of the observed dsDNA/ssDNA intermediates in Dna2-depleted cells had ssDNA branches ranging from ∼1000 to ∼5000 nucleotides, with some exceeding 10 000 nucleotides. Given that the strength of the DNA damage-checkpoint response is quantitatively related to the amount of RPA-bound ssDNA present in cells (70), these intermediates represent the most likely origin of toxic checkpoint signaling in cells devoid of Dna2. However, lagging-strand replication is intrinsically biased against the formation of DNA flaps greater than a few nucleotides (27,29,30) and extensive displacement synthesis on the lagging strand is limited by the inability of Pol δ to replicate through nascent nucleosomes (71,72). This leads us to suggest excessive Pif1-dependent RDR at stalled RFs as a source of dsDNA intermediates with extensive ssDNA branches when Dna2 is absent. Pol δ-mediated RDR can generate thousands of nucleotides worth of DNA (61). We envision that intrinsic D-loop instability and the passage of oncoming RFs may render a subset of nascent RDR strands permanently disengaged as RPA-bound ssDNA, eliciting a DNA damage-checkpoint response. In an attempt to reconcile the available data, we have included these molecular steps in a model of events at stalled RFs in the presence or absence of Dna2 (Figure 7).

We note that our model resolves a previously observed inconsistency (28) in explaining the essential nature of *DNA2* by a defect in Okazaki fragment processing. Pol δ-processivity on the lagging strand is redundantly stimulated by Pif1 and Rrm3 *in vivo*, arguing that removing Pif1 alone should not reverse the lethality of *dna2*Δ cells if this lethality resulted from strand-displacement DNA synthesis during Okazaki fragment maturation (28). In contrast, Pif1’s role in stimulating Pol δ-mediated DNA synthesis in the context of a D-loop during homologous recombination-coupled replication restart is non-redundant with Rrm3 (20). Thus, reduced RDR and checkpoint-activating by-products in the absence of Pif1 satisfyingly explains the restoration of viability to *dna2*-null cells. In conclusion, we propose that Dna2 is an essential gatekeeper at stalled RFs that promotes fork recovery and replication completion while at the same time suppressing inappropriate replication restart by RDR (Figure 7).

Mutations in *DNA2* that result in strongly reduced protein levels and/or dysfunction of DNA2’s helicase activity have been identified in patients with Seckel syndrome and microcephalic primordial dwarfism (7,10). A number of Seckel syndrome disease genes including *ATR*, *ATRIP*, *DONSON* and *TRAIP* have well-established roles in the response to RF-stalling (73–77), and their dysfunction provides an explanation for intrauterine and postnatal growth defects based on incomplete chromosome replication, cell-cycle arrest, and a general defect in cell proliferation during development (78). Our results reveal the essential nature of Dna2-dependent RF recovery, suggesting that *DNA2* falls into the same category of Seckel syndrome genes and that RF recovery defects in DNA2-deficient human cells (31,60) are directly disease-relevant. It will be interesting to explore whether a switch to inappropriate replication restart pathways contributes to the demise of human cells when DNA2 activity is perturbed. Finally, the observed overexpression of *DNA2* in cancer likely reflects an adaptation to increased levels of intrinsic RS, which is prominent in cancer cells (79). Its critical role in RF recovery supports *DNA2* as a therapeutic target (80,81), whose inhibition might generate clinical synthetic lethality in settings with elevated RS.

## Supplementary Material

gkaa524_Supplemental_File
