# Supplementary material for: Disease-associated DNA2 nuclease–helicase protects cells from lethal chromosome under-replication
Source: Nucleic Acids Res. 2020 Jun 16;48(13):7265–78. doi: 10.1093/nar/gkaa524 (PMC7367196; doi:10.1093/nar/gkaa524)
Supplement: gkaa524_Supplemental_File [file gkaa524_supplemental_file.pdf]

Supplementary Table S1. List of *Saccharomyces cerevisiae* strains used in this study.

Strain	Relevant genotype	Source
YRL19	*Mata, his∆3, leu2∆0, met15∆0, ura3∆0 (BY4741)*	GE Healthcare
CloneID 174	*YRL19, yen1::KAN*	GE Healthcare
YRL300	*YRL19, pif1::pif1-m2*	This study
YRL301	*YRL19, pif1::pif1-m2, yen1::KAN*	This study
YRL29	*YRL19, dna2::dna2-HD*	(1)
YRL302	*YRL19, dna2::dna2-HD, pif1::pif1-m2*	This study
YRL97	*YRL19, dna2::dna2-HD, yen1::KAN*	(1)
YRL303	*YRL19, dna2::dna2-HD, pif1::pif1-m2, yen1::KAN*	This study
YRL304	*YRL19, pif1::pif1-m2, pYES-DEST52 PIF1*	This study
YRL305	*YRL19, pif1::pif1-m2, pYES-DEST52 pif1-HD*	This study
YRL306	*YRL19, pif1::pif1-m2, pYES-DEST52 ccdB*	This study
YRL307	*YRL19, dna2::dna2-HD, pif1::pif1-m2, pYES-DEST52 PIF1*	This study
YRL308	*YRL19, dna2::dna2-HD, pif1::pif1-m2, pYES-DEST52 pif1-HD*	This study
YRL309	*YRL19, dna2::dna2-HD, pif1::pif1-m2, pYES-DEST52 ccdB*	This study
YRL310	*YRL19, dna2::HIS, pif1::pif1-m2*	This study
YRL311	*YRL19, dna2::HIS, pif1::pif1-m2, rad9::KAN*	This study
YRL316	*YRL19, dna2::HIS, pif1::pif1-m2, pAG416 GPD YEN1*	This study
YRL317	*YRL19, dna2::HIS, pif1::pif1-m2, mus81::NAT, pAG416 GPD YEN1*	This study
YRL318	*YRL19, dna2::HIS, pif1::pif1-m2, yen1::NAT, pAG416 GPD YEN1*	This study
YRL325	*YRL19, rad9::KAN*	This study
YRL326	*YRL19, dna2::HIS, rad9::KAN*	This study
YRL327	*YRL19, dna2::HIS, rad9::KAN, pAG416 YEN1*	This study
YRL328	*YRL19, dna2::HIS, rad9::KAN, yen1::NAT, pAG416 GPD YEN1*	This study
YRL330	*YRL19, dna2::dna2-ND/HD, pif1::pif1-m2*	This study
YRL334	*YRL19, pif1::pif1-R3E*	This study
YRL335	*YRL19, dna2::dna2-HD, pif1::pif1-R3E*	This study
YRL378	*YRL19, pif1::pif1-m2, rad9::KAN*	This study
YRL382	*YRL19, pif1::pif1-m2, pAG415 GPD YEN1-EGFP*	This study
YRL383	*YRL19, dna2 ::HIS, pif1::pif1-m2, pAG415 GPD YEN1-EGFP*	This study
YRL386	*YRL19, pif1::pif1-m2, pAG415 GPD YEN1-EGFP, pWJ1322 NOP1-dsRED*	This study
YRL387	*YRL19, dna2::HIS, pif1::pif1-m2, pAG415 GPD YEN1-EGFP, pWJ1322 NOP1-dsRED*	This study
YRL405	*YRL19, pif1::HIS*	This study
YRL408B	*YRL19, dna2::NAT, pif1::HIS*	This study
YRL411B	*YRL19, pif1::pif1-m2 pYES-DEST YEN1*^{ON}	This study
YRL412B	*YRL19, dna2::HIS pif1::pif1-m2 pYES-DEST YEN1*^{ON}	This study
YRL439B	*YRL19, dna2::dna2-HD, pif1::pif1-4a*	This study
YRL442B	*YRL19, dna2::dna2-HD, pif1::pif1-4a yen1::HIS*	This study
YRL444B	*YRL19, dna2::HIS, mus81::NAT, pif1::pif1-m2*	This study
YRL600	*YRL19, pif1::pif1-4a*	This study
YRL601	*YRL19, dna2::NAT, pif1::pif1-4a*	This study

1. Ölmezer, G., Levikova, M., Klein, D., Falquet, B., Fontana, G.A., Cejka, P. and Rass, U. (2016) Replication intermediates that escape Dna2 activity are processed by Holliday junction resolvase Yen1. *Nat. Commun.*, **7**, 13157.

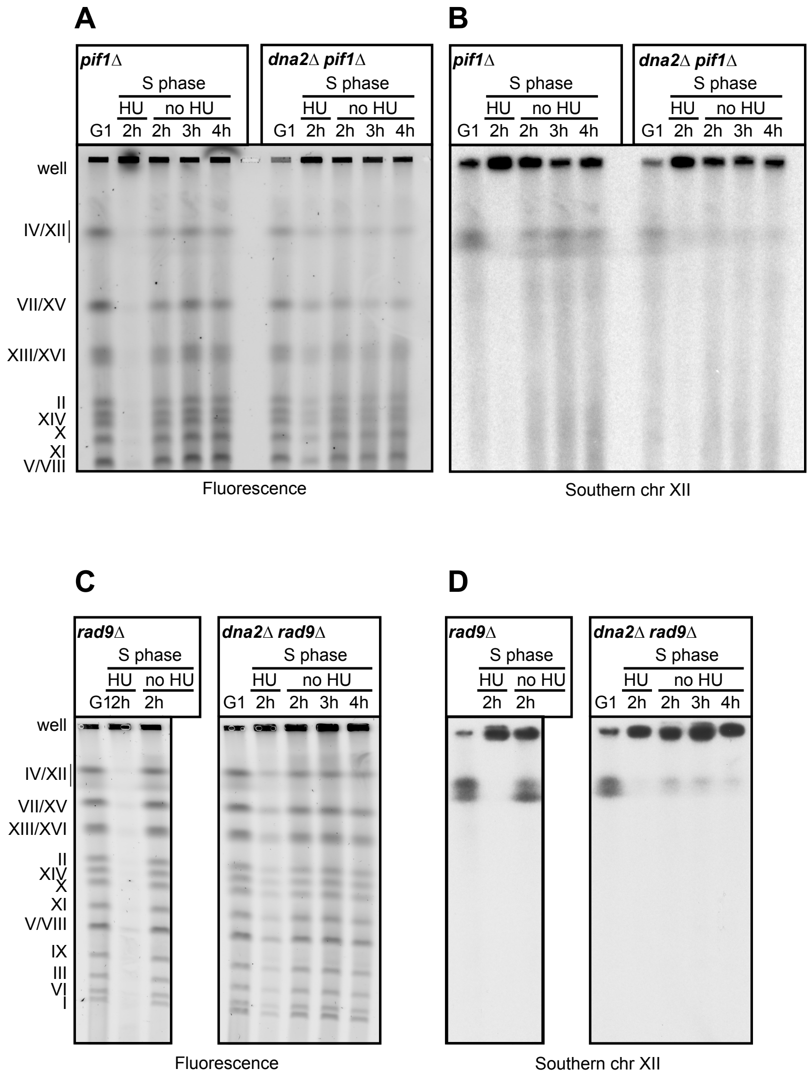

Figure S1. Dna2 is essential for replication completion regardless of the presence or absence of Pif1. (**A**) Representative PFGE run of *dna2*Δ *pif1*Δ and *pif1*Δ control samples; DNA stained with ethidium bromide. Gel-resolved DNA is labelled with chromosome numbers. G1-synchronized cells were released into medium containing 200 mM HU for 2 h, followed by drug wash-out and incubation in HU-free medium, which contained nocodazole to prevent mitosis. At the indicated experimental stages, genomic DNA was analysed for fully replicated chromosomes, which can be resolved through PFGE. (**B**) Southern blot analysis of a gel obtained as in panel A, probing for chromosome XII. A distinct shortfall in gel-resolved chromosome XII following transient RS-exposure is detected for the *dna2*Δ *pif1*Δ strain. (**C**, **D**) Representative PFGE run and Southern blot analysis as in panels A and B, respectively, performed with *dna2*Δ *rad9*Δ and *rad9*Δ control samples. A distinct shortfall in gel-resolved chromosome XII following transient RS-exposure is detected for the *dna2*Δ *rad9*Δ strain.

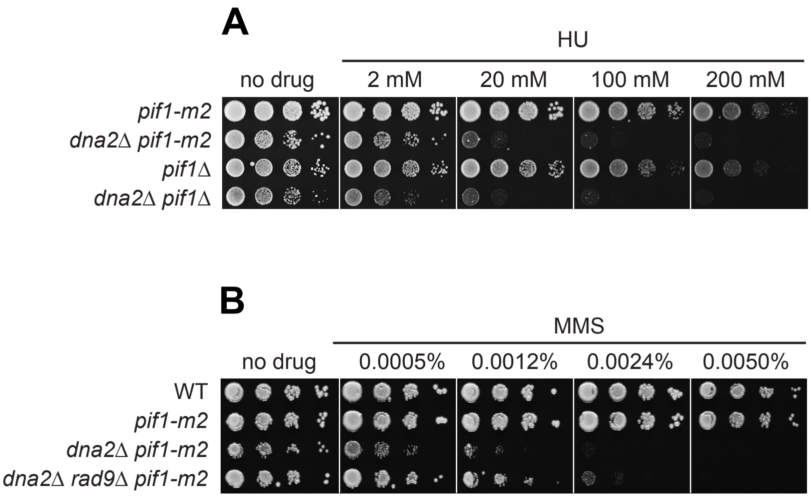

Figure S2. *dna2Δ* cells are sensitive to RS independently of Pif1 or Rad9 (**A**) Drop assays of serial dilutions of the indicated *pif1-m2* and *pif1Δ* mutant strains incubated on drug-free and HU-containing medium demonstrate the RS-sensitivity of *dna2Δ* cells, even in complete absence of Pif1. (**B**) Drop assays of serial dilutions of the indicated strains on drug-free and MMS-containing medium demonstrate the sensitivity of *dna2Δ* mutants to RS, even in absence of nuclear Pif1 or Rad9.

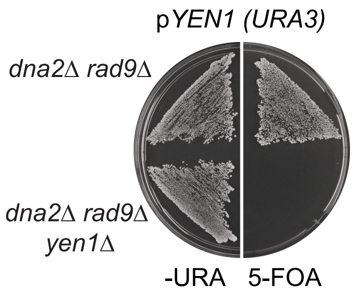

Figure S3. *YEN1* is essential in *dna2Δ rad9Δ* cells. Cells contained a Yen1-expressing plasmid (*pYEN1*). Lack of growth on 5-FOA indicates an inviable genotype.

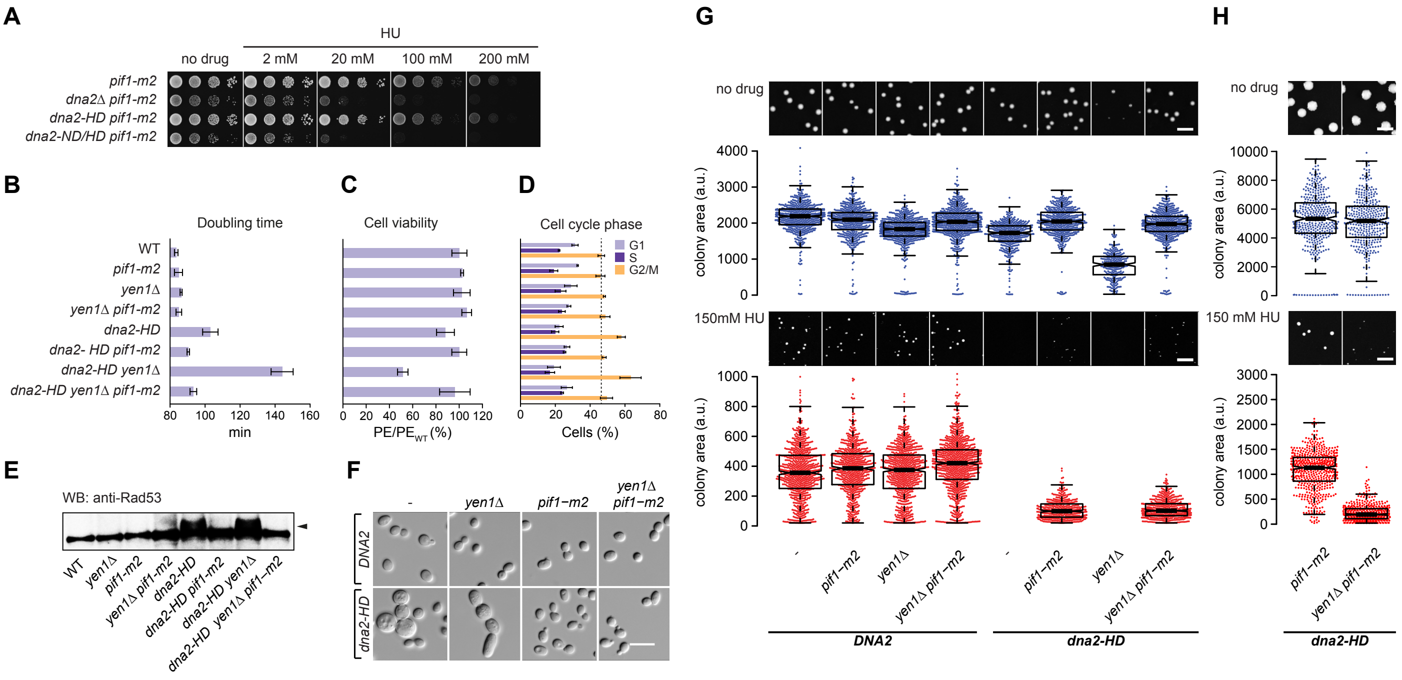

Figure S4. The nuclease activity of Dna2 protects Dna2 helicase-defective cells from RS. (**A**) Drop assays of serial dilutions of the indicated strains bearing different mutant alleles of *DNA2* in a *pif1-m2* background. *dna2-HD*, Dna2 R1253Q; *dna2-ND/HD*, nuclease/helicase-dead Dna2 with mutations D657A and R1253Q. (**B**) Doubling time measurements of the indicated strains presented as mean values ± SD (n = 3 independent experiments). (**C**) Cell viability of the indicated strains, assessed by plating efficiency (PE). Data represent mean values ± SD (n = 5 replicates), relative to wild-type (WT). (**D**) Cell-cycle phase distribution of the indicated strains, assessed by microscopic inspection of cell morphology, represented as mean percentage of cells in G1, S, and G2/M ± SD (n = 3 independent experiments). (**E**) Depletion of nuclear Pif1 suppresses chronic checkpoint activation in *dna2-HD* cells. Representative anti-Rad53 Western blots from whole-cell extracts of exponentially growing cultures of the indicated strains. Hyperphosphorylation of Rad53 (black arrowhead) indicates checkpoint activation. (**F**) Representative DIC images showing the cell morphologies observed for the indicated strains. Scale bar, 10 µm. (**G**) Colony-size measurements of cells grown on medium with or without 150 mM HU for two days, or (**H**) three days, with representative images. Scale bar, 5 mm. Box plots represent individual data points corresponding to single colonies (n > 250), median values (black rectangles), and the limits of the first and third quartile (lower and upper limits of the box, respectively).

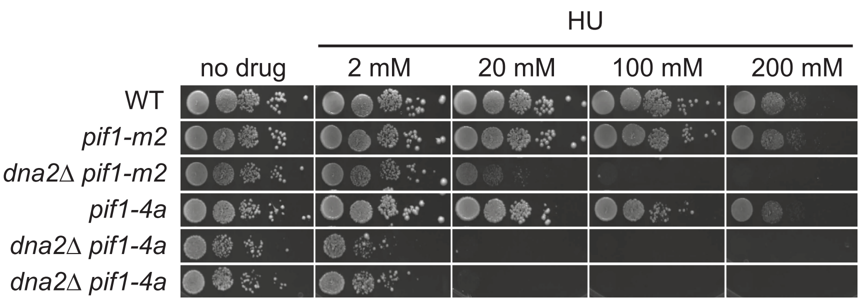

Figure S5. Disruption of the stress-responsive TLSSAES phosphorylation motif in Pif1 suppresses the lethality associated with loss of *DNA2*. Drop assays with the indicated strains on drug-free and HU-containing medium assessing growth and sensitivity to RS in the presence or absence of the *pif1-4a* allele at the endogenous *PIF1* locus in two independently created *dna2Δ pif1-4a* strains.
